# Clinical Activity and Tolerability of a 14-Day Infusional Ifosfamide Schedule in Soft-Tissue Sarcoma

**DOI:** 10.1155/2013/868973

**Published:** 2013-12-04

**Authors:** Juan Martin-Liberal, Salma Alam, Anastasia Constantinidou, Cyril Fisher, Komel Khabra, Christina Messiou, David Olmos, Scott Mitchell, Omar Al-Muderis, Aisha Miah, Mark Linch, Robin L. Jones, Michelle Scurr, Ian Judson, Charlotte Benson

**Affiliations:** ^1^Sarcoma Unit, The Royal Marsden Hospital, Fulham Road, London SW3 6JJ, UK; ^2^Department of Histopathology, The Royal Marsden Hospital, Fulham Road , London SW3 6JJ, UK; ^3^Department of Statistics, The Royal Marsden Hospital, Downs Road, Sutton SM2 5PT, UK; ^4^Department of Radiodiagnostics, The Royal Marsden Hospital, Fulham Road, London SW3 6JJ, UK; ^5^Spanish National Cancer Research Centre (CNIO), Clinical Research Programme, 3 Melchor Fernandez Almagro, 28029 Madrid, Spain; ^6^Department of Pharmacy, The Royal Marsden Hospital, Fulham Road, London SW3 6JJ, UK

## Abstract

*Background*. Soft-tissue sarcomas (STS) are a heterogeneous group of diseases with lack of effective treatments in most cases. Previous data suggest that continuous infusional ifosfamide regimens might improve cytotoxicity and tolerability compared to standard schedules. *Methods*. We retrospectively report the outcome of 35 patients affected by STS treated with a 14-day infusional ifosfamide regimen (1000 mg/m^2^/day) in our institution. Predictive factors for toxicity were also explored. *Results*. Median age was 53 years. There were 16 males and 19 females. Classification by histology was dedifferentiated liposarcoma (DDLPS): 22 (62.8%), synovial sarcoma: 7 (20%), myxoid/round-cell liposarcoma: 3 (8.5%), and others: 3 (8.5%). Overall, 7 patients (20%) achieved partial response (PR) and 10 patients (29%) achieved stable disease (SD). DDLPS showed special sensitivity: 5 patients (22.7%) had PR, 7 patients (31.8%) had SD, and disease control rate was 54.5%. Median progression-free survival and overall survival were 4.2 and 11.2 months, respectively. The most common toxicities were fatigue, nausea, and vomiting (all grades: 85.7%, 83%, and 54.3%, resp.). Neither hypoalbuminaemia nor gender was found to predict toxicity, although encephalopathy predominantly affected females. *Conclusion*. Ifosfamide administered as a 14-day continuous infusion is a safe regimen in STS with notable activity in DDLPS.

## 1. Introduction

Soft-tissue sarcomas (STS) are a group of rare tumours of mesenchymal origin. They encompass more than 50 different malignancies with different molecular features, clinical behavior, and prognosis. With respect to systemic therapy the most active agents achieve response rates of approximately 20–30% or less depending on the series [1, 2] and the median overall survival (OS) of patients with metastatic disease is only around 1 year [3]. With such disappointing data, the implementation of new active therapeutic strategies is an imperative.

One specific subtype of STS, dedifferentiated liposarcoma (DDLPS), responds especially poorly to chemotherapy with no reliably effective treatment being identified to date [4]. These tumours most commonly occur in the retroperitoneum. Primary surgical resection, where technically possible, is the mainstay of treatment, often involving extensive surgical procedures in specialist units, with en bloc multivisceral resections [5]. On development of disease recurrence further surgical excision may be performed after taking into account the disease extent and time since primary surgery but until recently treatment options in multiply recurrent, inoperable, or metastatic disease have been limited [6]. Experience with standard sarcoma chemotherapy regimens such as doxorubicin has been disappointing, although activity has been reported with trabectedin [7]. This intrinsic drug resistance makes the prognosis of a patient with advanced DDLPS very poor.

Ifosfamide may be used in the treatment of STS given either as a single agent [8] or in combination with doxorubicin [9]. The most commonly used regimen is three daily divided doses with doses ranging from 2 to 4 g/m^2^/day given as an inpatient over 4 hours. There is some evidence that a more prolonged infusion may have an improved cytotoxicity and tolerability profile although it is associated with worse urothelial toxicity compared with the three day infusion [10]. There is good stability data for the use of ifosfamide in disposable infusion pumps, used in the outpatient setting, and the longest stability data available is over a 9-day period [11–13]. Furthermore, continuous infusional ifosfamide is given in the outpatient setting and so is an attractive option to patients, lessening the impact on healthcare resources.

Since 2008, we have been using continuous infusional ifosfamide for patients with inoperable STS, especially DDLPS [13–15], at The Royal Marsden Hospital (United Kingdom).

This study assesses the toxicity and efficacy of infusional ifosfamide in all STS patients treated in our institution between September 2008 and December 2012.

## 2. Methods

### 2.1. Population

Clinical data from all STS patients who were treated with infusional ifosfamide in either the first or second line setting at The Royal Marsden Hospital from September 2008 to December 2012 were retrospectively collected. Cases were identified using the prospectively maintained Sarcoma Unit database and institutional ethical approval was obtained.

The patients all had histologically proven, inoperable, or metastatic progressing STS. Tumours were diagnosed according to the WHO Classification of Tumours of Soft Tissue and Bone [16]. The diagnosis of DDLPS was made where a pleomorphic component was found adjacent to well-differentiated liposarcoma or in intra-abdominal undifferentiated pleomorphic sarcomas that expressed Cyclin-Dependent Kinase 4 (CDK4) and p16 on immunohistochemistry and Mouse Double Minute 2 (MDM2) gene amplification using FISH. All patients were required to have adequate renal function as determined by either 24-hour creatinine clearance or EDTA, PS < 2, albumin > 30 g/L. The patients were treated with this infusional schedule because of histology, as there is some preliminary evidence to suggest activity in liposarcomas and synovial sarcoma.

### 2.2. Treatment

Continuous infusional ifosfamide was administered over 14 consecutive days in cycles of 28 days. The planned maximum number of cycles per patient was 6. Treatment was administered in an outpatient setting at a dose of 1000 mg/m^2^/day utilising a Baxter LV1.5 ambulatory pump, filled to a volume of 255 mL. Each pump was designed to infuse over 7 days and contained 7000 mg/m^2^ of ifosfamide. Mesna was also given to ameliorate urothelial toxicity. The total dose of mesna was equal to that of ifosfamide, either completely added to the pump or, depending on volume constraints, supplemented by oral tablets (rounded to the nearest measurable tablet). The oral dose of mesna was based on the assumption of a 2 : 1 oral to intravenous ratio. Patients attended hospital on day 8 for toxicity assessment and pump change. Every patient was given urine dipsticks and advised to test pH and blood with each void. Oral sodium bicarbonate 500 mg was used to alkalinise urine when urine dipstick showed pH < 6.5 and additional doses of mesna 1000 mg/m^2^ were to be taken if blood was present in the urine. Patients were advised to drink at least 1.5 litres of fluids daily to ensure an adequate diuresis. Also, thiamine 100 mg three times daily (tds) was prescribed to try and prevent encephalopathy and metoclopramide 10 mg tds was advised to be taken as required for nausea. Dose reduction of 20% in both ifosfamide and mesna was applied if the patient experienced grade 3-4 toxicity or persistent grade 2 toxicity.

### 2.3. Toxicity and Efficacy Evaluation

Toxicities were recorded and graded according to Common Terminology Criteria for Adverse Events (CTCAE) version 4.0 [17].

Computerized tomography (CT) scanning of the thorax, abdomen, and pelvis was performed at baseline and subsequently after every 2 cycles of treatment. Response assessment was evaluated according to RECIST criteria v 1.1 [18] by a named Sarcoma Unit radiologist. In the cohort of patients affected by DDLPS, the target lesions were selected by focussing on the dense dedifferentiated component in the knowledge that these areas behave most aggressively and are what the chemotherapy targets. Disease control was defined as partial response (PR) rate and stable disease (SD) rate.

Progression-free survival (PFS) was defined from date of starting treatment to date of objective progressive disease (PD) or death from disease. Any surviving progression-free patients were censored at last followup. OS was defined from date of starting treatment to date of death from any cause and any surviving patients were censored at last followup. Kaplan-Meier methods were used to calculate OS and PFS and the inverse Kaplan-Meier method was used to calculate median followup. SPSS version 21.0 was used to summarise any descriptive statistics and to determine OS and PFS.

## 3. Results

### 3.1. Patient Characteristics

A total of 35 consecutive patients received at least one cycle of continuous infusional ifosfamide between September 2008 and December 2012. The median age of the patients was 53 years (range 27–76 years) and there was a slight preponderance of females (54.3%). The predominant histology subtype was DDLPS (62.8%), followed by synovial sarcoma (20%). Two-thirds of patients were chemonaive and one-quarter had received a priorifosfamide-containing regimen. More detailed demographics and baseline characteristics are summarised in Table 1.

### 3.2. Toxicity

The total number of cycles of treatment administered was 35, with a median of 2 cycles given per patient (range 1–9). Twenty-six patients (74%) had to stop treatment early, while 9 patients (26%) reached 6 cycles. Among the patients who stopped treatment early, toxicity was the cause in 11 cases (42%), while 15 patients (58%) discontinued treatment due to disease progression. Dose reductions were necessary in 8 patients (22.8%) and 5 patients (14.3%) received only 1 cycle due to toxicity: grade 3-4 encephalopathy in 4 patients and 1 patient with gastrointestinal toxicity resulting in acute renal impairment secondary to dehydration.

All patients had toxicities attributable to the regimen. However, most side effects were mild (grade 1 or 2) and the majority of toxicities reported occurred from day 3 to day 12. The most common toxicities were fatigue (all grades, 85.7%), nausea (all grades, 83%), and vomiting (all grades, 54.3%).

For this regimen the most significant toxicity was encephalopathy with 12 patients (34.2%) having clinically significant symptoms, grade 3-4 in 6 patients. This led to discontinuation of treatment for 4 of these patients. Interestingly, all but one episode of encephalopathy occurred during the first treatment period. There is no data regarding the compliance of patients with the use of thiamine during treatment. There was one death in this cohort of patients, due to neutropenic sepsis; however overall myelotoxicity was acceptable with only 2 episodes of grade 3-4 myelosuppression for all cycles reported. Urothelial toxicity is an established toxicity for ifosfamide, with some reports suggesting prolonged infusions may increase this risk, but there was no significant urothelial toxicity seen, with no episodes of frank haematuria recorded. Renal toxicity was also mild with only 5 patients (14.3%) developing reversible grade 1-2 renal impairment. The toxicity data are summarized in Table 2.

Patient baseline demographics, renal function, and albumen level were analysed as potential predictors for toxicity, in particular encephalopathy, as this may allow for the identification of patients for whom a dose reduction may be useful in lessening the risk of developing significant encephalopathy. In this cohort baseline renal function, albumen, and gender were not statistically significant predictors for toxicity. Grade 3-4 encephalopathy did occur more frequently in females (5 versus 1 male) but due to sample size was not statistically significant.

### 3.3. Efficacy

Overall, 7 patients (20%) achieved PR and 10 patients (29%) achieved SD, in 7 cases lasting for more than 24 weeks. The median duration of response was 7 months (range 4–14) and the median duration of disease control (PR + SD) was 8.5 months (range 4–22). PD as best response was reported in 14 patients (40%), with 9 of these patients progressing during the first cycle of treatment. Among the patients that had previously received an ifosfamide-containing regimen, 1 SD and 2 PR were observed. Four patients had incomplete radiology for response assessment.

DDLPS proved to have particular sensitivity to the treatment: PR was seen in 5 out of 22 patients (22.7%) and 7 patients (31.8%) achieved SD, 5 of them (71.4%) for more than 24 weeks. In total, the disease control rate in this cohort was 54.5%. Activity was also seen in the synovial sarcoma cohort with 2 out of 7 patients achieving PR (28.6%) and a further patient had SD for 20 weeks. Amongst the other subtypes, 1 patient with myxoid liposarcoma out of 3 demonstrated SD and further disease stabilization was seen in 1 patient with pleomorphic sarcoma.

**Figure 1:**
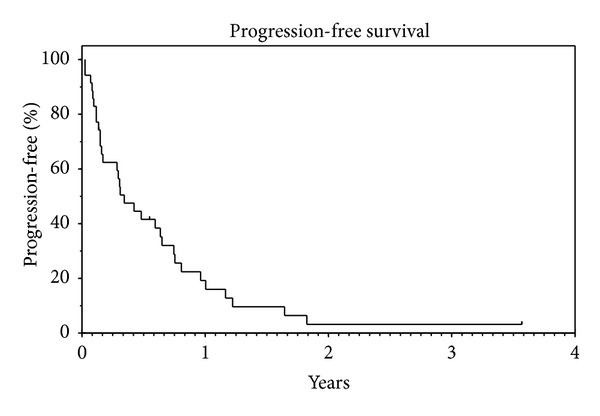
Median PFS: 4.2 months (95% CI: 2.0–6.3). PFS rate at 1 year: 19.2%. Median followup: 6.7 months.

**Figure 2:**
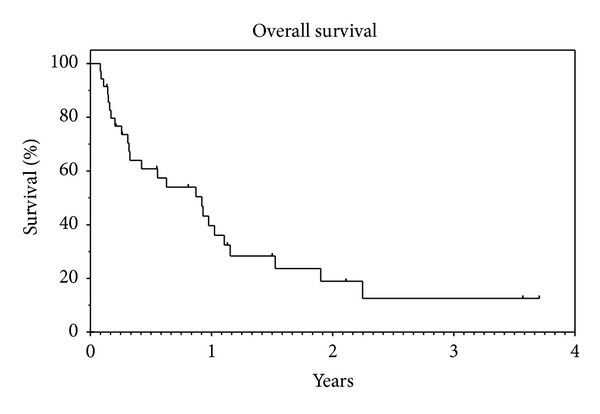
Median OS: 11.2 months (95% CI: 5.7–16.7). OS rate at 1 year: 39.6%. Median followup: 11.8 months.

In terms of survival, median PFS was 4.2 months (95% CI: 2.0–6.3) and PFS rate at 1 year was 19.2% with a median followup of 6.7 months (Figure 1). The median OS was 11.2 months (95% CI: 5.7–16.7) and 1 year OS rate was 39.6% with a median followup of 11.8 months (Figure 2). In the DDLPS cohort, median PFS was 3.7 months (95% CI: 0.0–7.8) and median OS was 10.5 months (95% CI: 3.3–17.6) with a median followup of 25.3 months.

**Figure 3:**
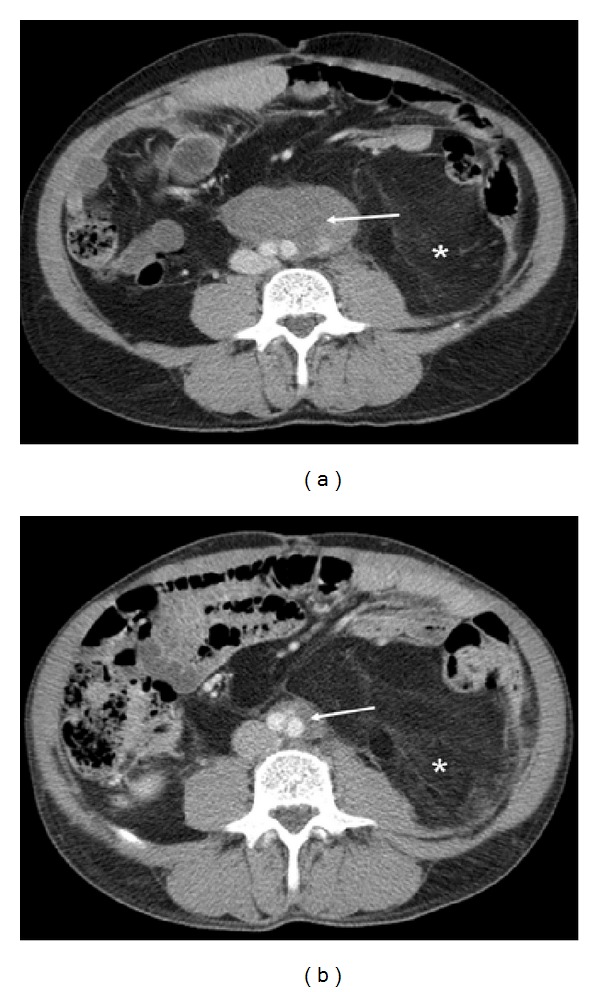
Axial contrast enhanced CT images of the abdomen in a 63-year-old male with retroperitoneal liposarcoma at baseline (a) and following 5 months therapy (b). Although the well-differentiated component filling the left side of the retroperitoneum (∗) showed little change, the dedifferentiated component (arrows) surrounding the aortic bifurcation reduced in size from 7.9 cm to 2.5 cm (PR).

**Figure 4:**
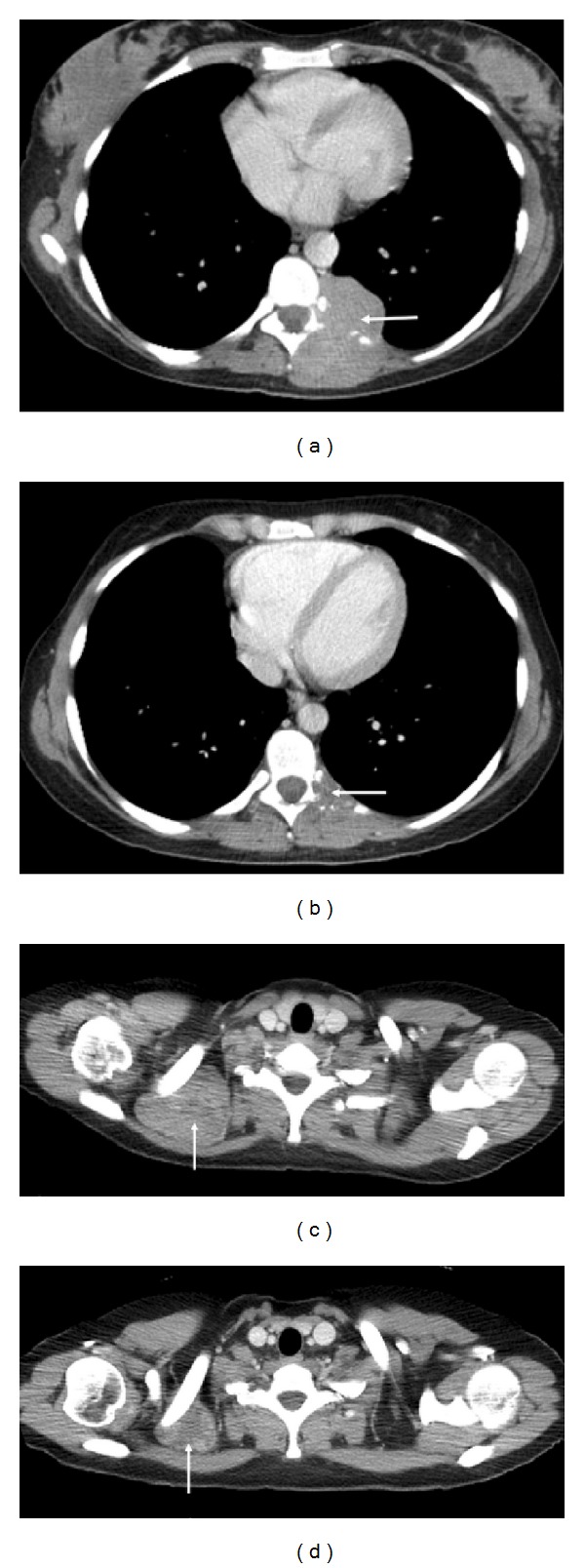
Axial contrast enhanced CT images of the lower and upper thorax in a 37-year-old female with metastatic synovial sarcoma at baseline ((a) and (c)) and following 2-month therapy ((b) and (d)). The 5.6 cm left sided paravertebral mass which was causing destruction of the posterior rib ((a), arrow) showed a dramatic reduction in size with only a thin plaque of indeterminate low attenuation tissue remaining at this site ((b), arrow). A further site of disease behind the right clavicle ((b), arrow) reduced in size from 6.5 cm to 3.6 cm ((d), arrow).

Radiological responses are illustrated in Figures 3 and 4.

## 4. Discussion

Our report demonstrates that ifosfamide administered as a 14-day continuous infusion is a safe outpatient regimen for STS. The majority of toxicities observed were mild and easily manageable and usually appeared within the first few days of treatment, allowing early intervention with effective symptomatic treatment. Haematuria did not appear to be a significant toxicity with this regimen and the rate of clinically significant neutropenia was acceptable. Moreover, this regimen has also the advantage of not requiring planned inpatient hospital admission. This has a direct impact on the quality of life of patients, especially important as this treatment regimen is usually given in the palliative setting.

There was a higher than expected rate of encephalopathy in our cohort, which is a potential concern for an outpatient regimen. Although not statistically significant, we found that female gender was a predictor for developing encephalopathy. One possible explanation might be the intrinsic pharmacokinetic difference between genders. An increased CYP3A4 expression in females may explain the differences in sex-dependent drug clearance [19]. These differences had no consequences on the ifosfamide 4-hydroxylation activities of liver microsomes *in vitro*. In contrast, in the ifosfamide N-dechloroethylation reaction, a statistically significant difference between the liver microsomes of male and female patients was found [20]. The relative greater production of chloroacetaldehyde in the female liver might explain the relative increase in neurotoxicity observed. Whatever the reason, delivery of cycle one at a lower dose (for instance, 750 mg/m^2^/day) and dose escalation should be taken into consideration if treatment is tolerated in female patients, given the increased risk of encephalopathy observed. Interestingly, this toxicity profile differs from the one previously reported by Meazza et al. [12]. In their paediatric population, only 4 out of 14 patients experienced mild nausea, and antiemetic prophylaxis was required in only a minority of patients. Moreover, no neurological or renal toxicity was observed, which illustrates the different tolerance to this treatment in adults.

Our regimen also showed evidence of antitumour activity, particularly in DDLPS, synovial sarcoma, and myxoid/round-cell liposarcoma. Synovial sarcoma is known to be particularly sensitive to ifosfamide, even upon rechallenge [21]. In contrast, DDLPS is not sensitive to conventional chemotherapy, although trabectedin has shown some activity [7]. Investigational novel drugs such as inhibitors of MDM2 or CDK4 have recently been assessed in this histological subtype with encouraging results [22, 23]. Our efficacy data show that infusional ifosfamide might be a treatment option especially in DDLPS, with a disease control rate of 54.5% in patients with confirmed PD prior to starting treatment.

In conclusion, our data suggest that infusional ifosfamide is a feasible schedule in STS. However, due to the retrospective nature and the small patient numbers of our report, prospective validation is clearly required. The European Organization for Research and Treatment of Cancer (EORTC) is planning to conduct a prospective randomized phase II trial that will compare infusional ifosfamide with cabazitaxel in patients affected by DDLPS. This study will hopefully provide us with more reliable information in a near future.

## 5. Conclusions

Infusional ifosfamide is a safe treatment in STS, with notable activity in dedifferentiated liposarcoma. This regimen deserves further investigation.

## Figures and Tables

**Table 1:** Patient characteristics.

Total	35
Age (median, range)	53 (27–76)

	*n* (%)

Sex	
Male	16 (45.7%)
Female	19 (54.3%)
ECOG performance status at baseline	
0	1 (2.8%)
1	30 (85.7%)
2	4 (11.4%)
Histology	
De-differentiated liposarcoma	22 (62.8%)
Synovial sarcoma	7 (20%)
Myxoid/round-cell liposarcoma	3 (8.5%)
Other	3 (8.5%)
Previous chemotherapy	
Chemotherapy naive	23 (65.7%)
Ifosfamide-containing regimen	9 (25.7%)
Nonifosfamide-containing regimen	3 (8.5%)
Previous surgery	
Yes	23 (65.7%)
No	12 (34.3%)
Baseline creatinine clearance (Cockroft-Gault formula)	
Median	97 mL/min (51.9–215.8)
Baseline albumin	
Median	35 g/L (21–43)

**Table 2:** Toxicity—number of patients.

Toxicity	Grade 1-2	Grade 3-4	Total
Fatigue	28 (80%)	2 (5.7%)	30 (85.7%)
Nausea	24 (68.6%)	5 (14.3%)	29 (83%)
Vomiting	16 (45.7%)	3 (8.6%)	19 (54.3%)
Myelosuppression	14 (40%)	2 (5.7%)	16 (45.7%)
Encephalopathy	6 (17.1%)	6 (17.1%)	12 (34.2%)
Constipation	10 (28.6%)	0	10 (28.6%)
Diarrhoea	4 (11.4%)	2 (5.7%)	6 (17.1%)
Renal function impairment	5 (14.3%)	0	5 (14.3%)
Liver function tests alteration	1 (2.8%)	0	1 (2.8%)
